# Identification and validation of a gene-based signature reveals SLC25A10 as a novel prognostic indicator for patients with ovarian cancer

**DOI:** 10.1186/s13048-022-01039-4

**Published:** 2022-09-16

**Authors:** Qi-jia Li, Juan Wang, Jing Jiang, Bing Lin

**Affiliations:** 1 Hospital of Chengdu University of Traditional Chinese Medicine, No. 39 Shi-er-qiao Road, Chengdu, 610072 Sichuan Province China; 2 Department of Public Health, School of Clinical Medicine, Chengdu University of Traditional Chinese Medicine, Chengdu, 610072 China

**Keywords:** Ovarian Cancer, Risk Score, Prognosis, SLC25A10

## Abstract

**Background:**

Ovarian cancer is a common gynecological cancer with poor prognosis and poses a serious threat to woman life and health. In this study, we aimed to establish a prognostic signature for the risk assessment of ovarian cancer.

**Methods:**

The Cancer Genome Atlas (TCGA) dataset was used as the training set and the International Cancer Genome Consortium (ICGC) dataset was set as an independent external validation. A multi-stage screening strategy was used to determine the prognostic features of ovarian cancer with R software. The relationship between the prognosis of ovarian cancer and the expression level of SLC25A10 was selected for further analysis.

**Results:**

A total of 16 prognosis-associated genes were screened to construct the risk score signature. Survival analysis showed that patients in the high-risk score group had a poor prognosis compared to the low-risk group. Accuracy of this prognostic signature was confirmed by the receiver operating characteristic (ROC) curve and decision curve analysis (DCA), and validated with ICGC cohort. This signature was identified as an independent factor for predicting overall survival (OS). Nomogram constructed by multiple clinical parameters showed excellent performance for OS prediction. Finally, it’s found that patients with low expression of SLC25A10 generally had poor survival and higher resistance to most chemotherapeutic drugs.

**Conclusions:**

In sum, we developed a 16-gene prognostic signature, which could serve as a promising tool for the prognostic prediction of ovarian cancer, and the expression level of SLC25A10 was tightly associated with OS of the patients.

## Background

Ovarian cancer is one of most lethal cancers for women worldwide, characterized by its malignant aggressiveness and poor prognosis [1, 2]. According to the data of GLOBOCAN 2020, the incidence of ovarian cancer is about 1.6%, with a mortality rate of 2.1% [3]. The 5-year survival rate of ovarian cancer is less than 30%, despite of advanced treatment with the combination of targeted therapy, chemotherapy and radiotherapy [4, 5]. The overall survival (OS) of ovarian cancer is highly dependent on disease stages; however, at initial diagnosis, more than 75% of ovarian cancer patients are stage III or IV [6], due to the fact that the early phases are asymptomatic [7]. Therefore, the pressing question remains on improving the prognosis of ovarian cancers.

Accumulating evidence established that the prognosis of ovarian cancer is correlated with a variety of factors, including histological types, pathological stages, age and immune status [8, 9]. In practice, current methods for prognostic evaluation are still invasive and non-systematic. Alternatively, prognostic modeling of ovarian cancer is of clinical significance for assessment of risk factors, to develop novel biomarkers [10]. In terms of prognostic modeling, it’s possible to estimate the OS and risk of recurrence. Moreover, prognostic modeling could also pave the way for identification of subgroups of patients with unfavorable prognosis, for individualize clinical interventions [11]. With rapid progression of high-throughput sequencing technologies (e.g., genomic and transcriptomic sequencing), an increasing number of key driver genes in the initiation and progression of ovarian cancers have been discovered [7, 12, 13], which provides the opportunity to build prognostic models for ovarian cancers.

In this study, we investigated differentially expressed genes (DEGs) in ovarian cancers by exploring TCGA (*The Cancer Genome Atlas*) database [14], and applied interrogated bioinformatics approaches to screen genes and identified prognostic signatures associated with the survival of ovarian cancer patients. The efficiency of the signature was evaluated at multiple levels, and external validation was performed using the ICGC database (The International Cancer Genome Consortium) [15]. In addition, we constructed a nomogram with satisfactory credibility for ovarian cancer patients. Moreover, we identified SLC25A10, a gene encodes a mitochondrial dicarboxylate transporter [16], as an excellent prognostic marker and potential therapeutic target for ovarian cancers. Taken together, our study described the prognostic signature for ovarian cancers, and provided novels targets for the improvement of patient prognosis.

## Results

### Identification of the prognostic signature by risk scores in ovarian cancers.

**Fig. 1:**
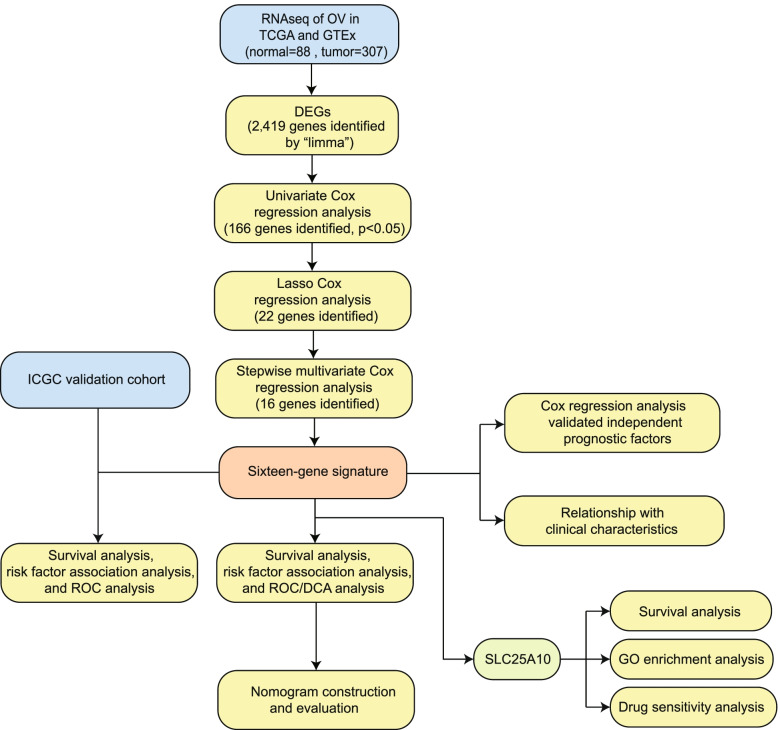
The workflow chart of this study. A flow chart of the systematic identification and validation of the 16-gene signature for the prognostic prediction of ovarian cancer

To explore the prognostic signature in ovarian cancers, we analyzed the datasets of ovarian cancer samples from TCGA database (307 cases), in parallel with non-tumor samples (88 cases) from GTEx. After conducting univariate Cox regression, LASSO-COX regression, and multivariate Cox regression analyses, we established the gene signature (Fig. 1).

**Fig. 2:**
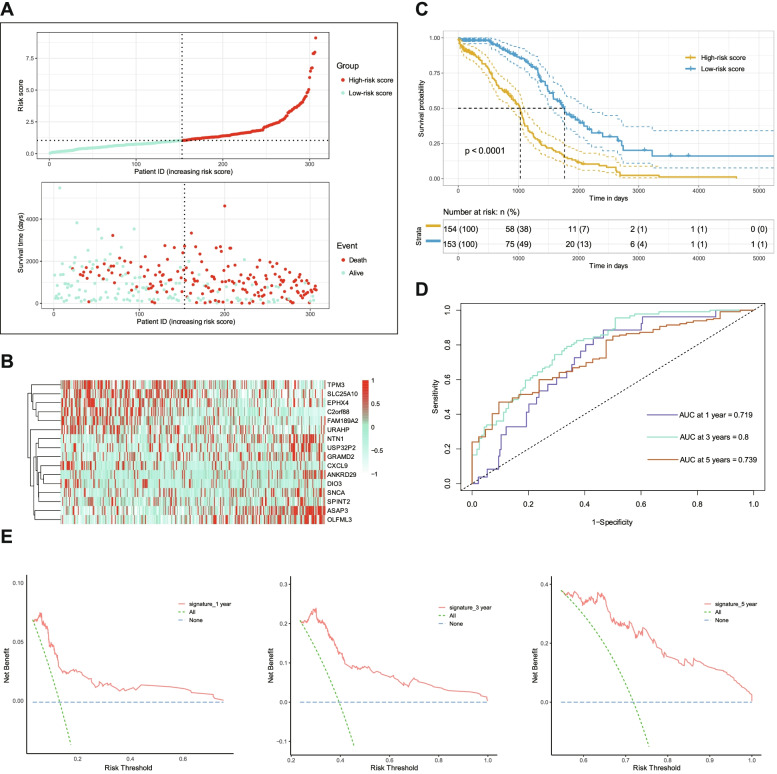
Identification of risk score based on gene signature of patients with ovarain cancer in TCGA. **A** Risk plot of each point sorted based on risk score, representing one patient. Green and red points represent patients with low- and high-risk scores, respectively. **B** Distribution of risk score and significant genes of ovarian cancer in TCGA. **C** Kaplan–Meier analysis of ovarian cancer patients was stratified by median risk in TCGA. High risk scores are associated with poor survival. **D** ROC curves of risk score in prognosis prediction of ovarian cancer in TCGA. **E** DCA curves of risk score in prognosis prediction of ovarian cancer in TCGA

We distinguished patients by low- and high-risk scores, and established the association maps, showing that the mortality of ovarian cancer patients increased as the curve of risk score grew (Fig. 2A). Then, we screened 16 related genes to create a prognostic signature comprising of TPM3, SLC25A10, EPHX4, C2orf88, FAM189A2, URAHP, NTN1, USP32P2, GRAMD2, CXCL9, ANKRD29, DIO3, SNCA, SPINT2, ASAP3 and OLFML3 (Fig. 2B). It’s noted that genes including TPM3, SLC25A10, EPHX4, C2orf88, FAM189A2 and URAHP were highly expressed in the group of low-risk scores, whereas other genes in the group of high-risk scores (Fig. 2B).

We next performed Kaplan-Meier logarithm test to assess the OS difference between different risk score groups. Results showed that patients with high-risk scores had worse survival than those with low-risk scores (Fig. 2C). The receiver operating characteristic (ROC) curves demonstrated the predictive efficiency of the OS-related signature. The area under the curve (AUC) of 1-, 3- and 5-years was 0.719, 0.800 and 0.739, respectively, indicating an excellent predictive value of the signature (Fig. 2D). Moreover, the DCA (decision curve analysis) results showed that our prognostic model exhibited good net benefit for 1-, 3- and 5-years (Fig. 2E). Altogether, ROC and DCA demonstrated that our 16-gene signature performed well in predicting both short-term survival (1-year and 3-year) and long-term survival (such as 5-year) for patients with ovarian cancers.

### Risk score is an independent prognostic marker of ovarian cancers

**Fig. 3:**
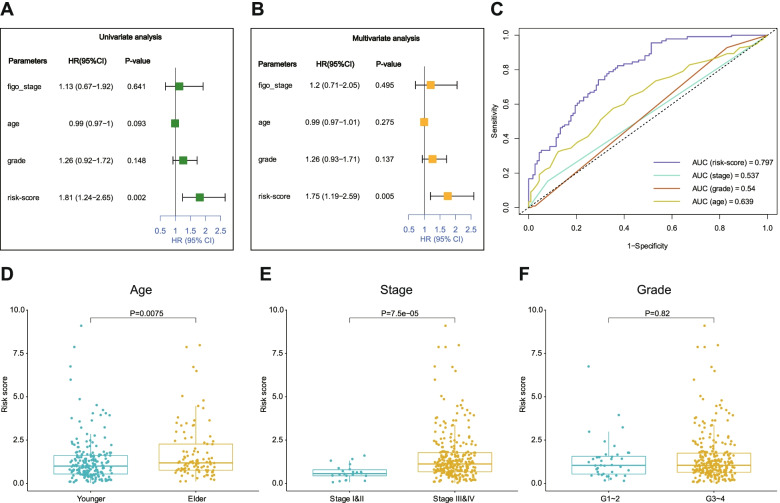
Characterization of prognostic signature in ovarian cancer of TCGA. (A, B) Univariate (**A**) and multivariate (**B**) Cox proportion hazard regression for OS of ovarian cancer in training group of TCGA. **(C)** Multi-index ROC curve of risk score and other indicators for predictions of survival time in ovarian cancer patients. **(D-F)** Boxplots showing the distribution of risk score in ovarian cancer samples stratified by different factors, including age (**D**), stage (**E**) and grade (**F**)

To investigate whether the risk score was an independent indicator of prognosis, we adopted to clinical stages, age, pathological grade and risk scores for univariate and multivariate Cox regression analysis. Results showed that only the risk score (HR=1.81/1.75, 95% CI=1.24-2.65/1.19-2.59, *p*=0.002/0.005) was significantly associated with OS of ovarian cancer (Fig. 3A, B), suggesting that the risk score could be an independent factor to predict OS of ovarian cancer. Moreover, ROC curves were drawn to compare the predictive accuracy of above parameters, revealing that the AUC of the risk score was larger than that of other clinical characteristics (Fig. 3C).

To further validate the clinical significance of our prognostic signature, we assessed the association between the risk score and clinicopathological variables. It was found that there was a statistically significant difference in risk scores between age > 65 years (elder) and age ≤ 65 years (younger) (*P*=0.0075) (Fig. 3D), as well as the pathological stages III&IV vs. pathological stages I&II (P=7.5e-05) (Fig. 3E). It’s also noted that the risk scores for histological grades G3-4 were not significantly different from G1-2 (*P*=0.82) (Fig. 3F).

### Validation of the prognostic signature in an independent cohort

**Fig. 4:**
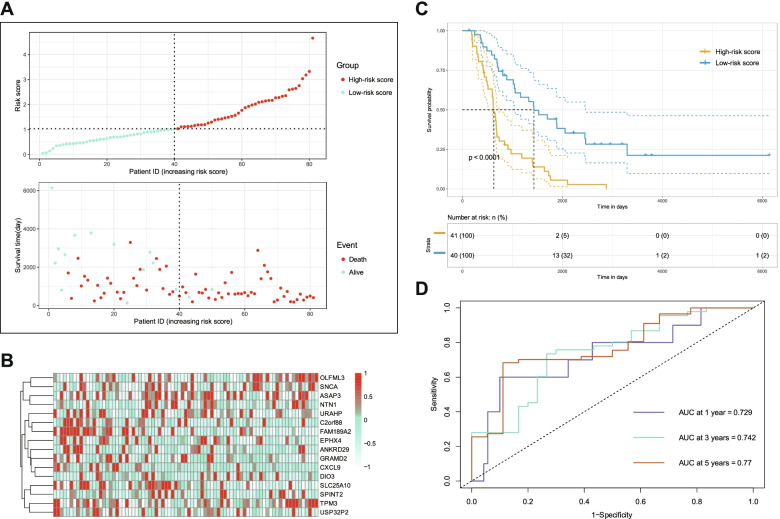
Validation of risk score based on gene signature of patients with ovarain cancer in ICGC. **A** Risk plot of each point sorted based on risk score, representing one patient. Green and red points represent patients with low- and high-risk, respectively. **B** Distribution of risk score and patient survival time of ovarian cancer in ICGC. **C** Kaplan–Meier analysis of ovarian cancer patients was stratified by median risk in ICGC. **D** ROC curves of risk score in prognosis prediction of ovarian cancer in ICGC

To confirm the prognostic signature in an independent external dataset, we calculated the risk score for each patient in the ovarian cancer dataset of ICGC using the same formula. As anticipated, the association maps showed consistent results with the training set, revealing that genes including SLC25A10, C2orf88 and FAM189A2 were enriched in the group of low-risk scores, and OLFML3 in the group of high-risk scores (Fig. 4A, B).

Subsequent Kaplan–Meier analysis verified that the low-risk score group still displayed better OS than the high-risk score group (Fig. 4C). Additionally, the ROC results showed that the AUC of 1-, 3- and 5-years was 0.729,0.742 and 0.77, respectively (Fig. 4D). All these results confirmed the excellent survival predictive ability of our prognostic signature.

### Establishment of the nomogram prognostic model

**Fig. 5:**
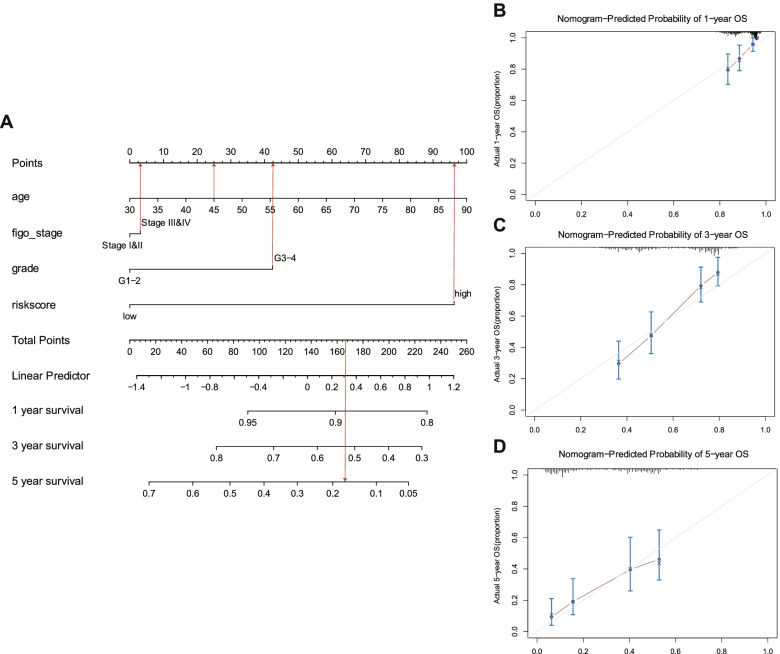
Contruction of a nomogram to predict the patient survival in ovarian cancer. **A** The nomogram using age, stage, grade and risk score to predict the OS of patients with ovarian cancer in TCGA. **B-D** The calibration plot to evaluate the nomogram. Y-axis, actual survival. X-axis, predicted survival of 1-year (**B**), 3- year (**C**), and 5-year (**D**), respectively. The solid line represents the predicted nomogram

To provide better prognosis for ovarian patients based on our risk score model, we conducted a nomogram to predict the OS of 1-, 3-, and 5-years using four prognostic factors, including clinical stages, age, pathological grade, and risk scores (Fig. 5A). The C-index of the nomogram was 0.71, which showed moderate accuracy. Results of the calibration curves showed that the predicted calibration curves were quite close to standard curves (the diagonal), implying that the nomogram is robust in the OS prediction for ovarian cancers (Fig. 5B-D).

### The relationship between SLC25A10 and prognosis

As demonstrated in the training set (Fig. 2) and testing set (Fig. 4), we noticed that only SLC25A10, C2orf88, FAM189A2 and OLFML3 displayed consistent expression patterns. Among all these four genes, SLC25A10 showed the most dramatically alterations, which may serve as a candidate for predicting the prognosis of ovarian cancer patients.

**Fig. 6:**
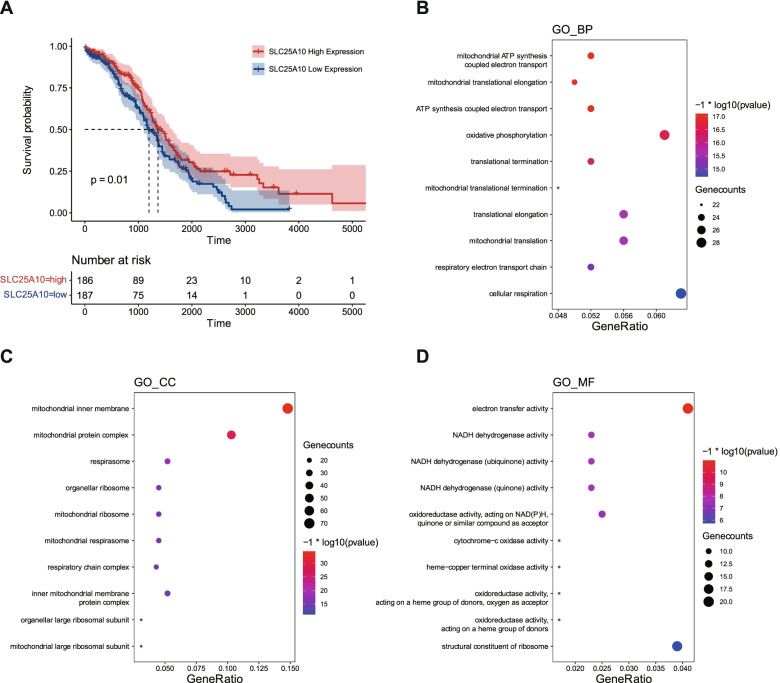
The correlation of SLC25A10 with the overall survival and molecular functions in patients with ovarian cancer. **A** Kaplan–Meier survial analysis of SLC25A10 with ovarian cancer using the information from TCGA dataset. Patients are divided into low and high SLC25A10 groups by median expression level. **B-D** Results of GO analysis showing the consistently altered gene profiles with SLC25A10 in TCGA dataset, including biological process (**B**), cellular component (**C**) and molecular function (**D**)

To test this hypothesis, we analyzed the OS of ovarian cancer patients based on SLC25A10 expressions. Results showed that low expression of SLC25A10 was associated with the poor survival time in ovarian cancer patients from TCGA dataset (Fig. 6A). To further understand how SLC25A10 is involved in ovarian cancers, we employed GO analysis to figure out SLC25A10-associated pathways. We identified 578 genes that are associated with SLC25A10 based on the criteria of correlation value > 0.3 and p < 0.05. The GO analysis indicated that the significant GO terms were enriched in mitochondria-dependent events, including ATP productions, mitochondrial protein synthesis, respirasome assemble and NADH dehydrogenase activity (Fig. 6B-D). These top-ranked metabolic pathways strongly support the notion that SLC25A10 was the mitochondrial dicarboxylate carrier to fueled TCA cycle and energetics [17, 18].

**Fig. 7:**
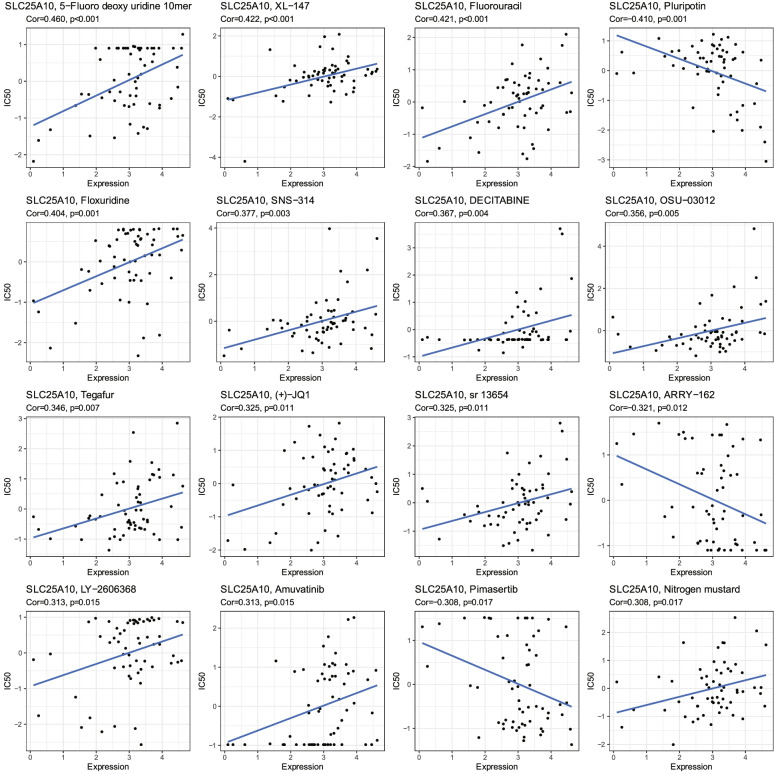
The sensitivity analysis of SLC25A10 and multiple chemotherapeutic drugs. The top 16 drugs with high correlation with SLC25A10 expression were demonstrated

Based on the findings that higher SLC25A10 was associated with better prognosis in ovarian cancer patients, we assumed that elevated SLC25A10 expression may be beneficial to improve the overall survival time of patients. To facilitate better and more precise treatment, we assessed the impact of SLC25A10 on drug sensitivity based on the CellMiner database (http://discover.nci.nih.gov/cellminer/) [19]. The NCI-60 database containing 60 different cancer cell lines from nine different types of tumors was accessed through the CellMiner interface. Drugs used in this sensitivity analysis includes 216 drugs approved by FDA and 574 drugs tested by clinical trials. The drug sensitivity was measured by the z-score, and higher scores implied that cells are more sensitive to drug treatment. After Pearson correlation analysis, the top 16 interconnections of SLC25A10-drugs were demonstrated, showing that elevated SLC25A10 was positively associated with increased cellular sensitivity to most chemotherapeutic drugs, except for a few, such as Pluripotin, ARRY-162 and Pimasertib, which were more sensitive to low SLC25A10 cells (Fig. 7).

## Discussion

Currently, the treatment of ovarian cancer is mainly based on surgery and chemotherapy [20, 21]. And emerging clinical and fundamental studies has established the cellular and molecular signatures of ovarian cancers [22, 23]. In practice, however, nearly 80% of patients with ovarian cancers were diagnosed only after the onset of symptoms when the disease has progressed to an advanced stage. Therefore, there is a necessity to seek novel biomarkers to predict the outcome of ovarian cancer, which can be used to develop targeted therapies for ovarian cancer, especially in its early stages. As a progressive disease, ovarian cancer requires reliable biomarkers to predict prognosis. However, studies on prognostic models in ovarian cancer were limited [24–26], and the pressing question remains on predicting ovarian cancer outcomes [27]. Identification of novel genetic features that predicts patient prognosis may facilitate the choice of treatment options and improve patient survival and life quality.

In this study, by analyzing datasets of RNA sequencing from the TCGA database, we developed a risk score model using 16 genes to predict the prognosis of ovarian cancer patients and validated the prediction of the model using an external dataset. The risk score was demonstrated to be an independent prognostic factor by univariate and multivariate analyses. In addition to traditional clinicopathological indicators, risk scores based on prognostic models could be applied clinically to provide convenient and better prognostic monitoring. Therefore, our work supplements current understandings on the prognostic prediction in ovarian cancers. Although our results exhibit relatively promising predictive ability, *in vitro* and *in vivo* experiments are still needed to further validate our results.

In addition to the identification of the risk score model, we also pinpointed that SLC25A10 may be a good prognostic marker and therapeutic target for ovarian cancers. This gene encodes a member of a family of proteins that translocate small metabolites across the mitochondrial membrane, and thus may be involved in the colon formation and migration of cancer cells. Several studies demonstrated that SLC25A10 expression is upregulated in a variety of tumors compared to normal tissues [28]. In 13/20 tumor types, SLC25A10 exhibited >2-fold increase in cancer expression as compared to stromal cells. Moreover, SLC25A10 may play an oncogenic role in human osteosarcoma, and high SLC25A10 expression was associated with poor clinicopathological parameters [29]. In this study, we found that SLC25A10 expression was associated with low-risk score of ovarian cancers. Moreover, the Kaplan-Meier analysis further confirmed that high SLC25A10 expression was correlated with better prognosis for ovarian cancer patients. Interestingly, the gene-drug analysis showed that the sensitivity of ovarian cancer tumor cells to most chemotherapeutic agents was also diminished by low SLC25A10 expressions; however, Pluripotin, ARRY-162 and Pimasertib were more sensitive to low SLC25A10 cells. These findings provide novel insights into the precise chemotherapeutic strategy for distinct ovarian cancer patients by different SLC25A10 expressions.

## Conclusions

In summary, our study identified and validated a promising 16 gene prognostic signature to predict the clinical outcomes for ovarian cancer patients. Moreover, we pinpointed that SLC25A10 as a novel indicator for prediction of patient prognosis in ovarian cancers, and provided potential chemotherapeutic strategy for tumor treatment. Our work not only reveals the molecular signature of ovarian cancers, but also contributes to the tumor prognosis and treatment.

## Methods

### Data acquisition and construction of prognostic signature

The dataset of tumor samples of ovarian cancer was obtained from TCGA database (https://portal.gdc.cancer.gov/), and para-cancer normal samples from Genotype-Tissue Expression Portal (GTEx) (https://xenabrowser.net/), which were combined as the training set. The tumor samples from ICGC database (https://daco.icgc.org/) were taken as the validation set.

To establish the gene signature, 2,419 differentially expressed genes (DEGs) between ovarian cancer samples (TCGA, *n*=307) and non-tumor samples (GTEx, *n*=88) were screened by limma-voom analysis of R software (version 4.0.3), with the p-value < 0.05 and Log2(Fold Change)>2. After the univariate COX regression analysis with the *P*-value < 0.05, 166 genes were included in the LASSO-COX regression analysis. Finally, by step wise multivariate COX regression analysis and weighting the estimated cox regression coefficients, 16 genes were obtained to construct a risk score signature for prognostic analysis. Specifically, the univariate COX regression analysis was performed by the “survival” R package. The LASSO-COX regression analysis was performed by the “glmnet” R package [30]. The step wise multivariate COX regression analysis was performed by the “survival” R package [31]. A risk score formula to calculate the probability of inferior survival for each sample was constructed based on the expression of prognostic genes in multivariate Cox-regression analysis, weighted by coefficients. We calculated the risk score of each patient according to the following formula:

$$\mathrm{Risk}\ \mathrm{score}=\mathrm{expression}\ \mathrm{of}\ \mathrm{gene}\ 1\times \beta 1+\mathrm{expression}\ \mathrm{of}\ \mathrm{gene}\ 2\times \beta 2+\cdots +\mathrm{expression}\ \mathrm{of}\ \mathrm{gene}\ \mathrm{n}\times \beta \mathrm{n}$$

### Survival analysis

To assess the overall survival (OS) rates, we enrolled 307 ovarian cancer patients and divided them into two subgroups based on the median level of the risk score as a cutoff, with those greater than or equal to the median level being the high-risk group and those less than the median level considered as low-risk. Risk factor association maps were created using risk scores and visualized by “ggplot2” and “pheatmap” R packages. Survival analysis was performed utilizing the “survival” and “survminer” R packages (“survfit” and “ggsurvplot” function) to evaluate the variance in survival status between the high-risk and low-risk groups, to confirm the validity and robustness of the OS risk prognostic signature. The probability of OS was estimated by the Kaplan-Meier method. The significance of the difference in the probability of OS between the different groups was measured by the log-rank test with a threshold of p-value < 0.05. The R package “timeROC” was used to construct ROC curves for assessment of the prognostic signature, and the corresponding area under the ROC curve (AUC) was measured to estimate the sensitivity and specificity. To better demonstrate the predictive performance of the model, decision curve analysis (DCA) were also employed to validate the prognostic of the signature [32]. Additionally, univariate and multivariate cox analyses were performed to determine the independence of the risk score in prognosis estimation, results of which were visualized by “forestplot” R package. The correlations between risk scores and clinical characteristics (age, clinical stage, and pathological grade) were visualized by the “ggpubr” R package.

### External dataset validation

The same formula was applied to calculate risk scores for ovarian cancer dataset in ICGC, and the samples were also divided into two subgroups of high and low risks using the median value as a cutoff, then Kaplan-Meier survival curve analysis and risk factor association maps were created. Similarly, ROC curve analysis was performed to assess the effectiveness of OS predictions.

### Construction of the nomogram

Prognostic nomograms for predicting the likelihood of 1-year, 3-year and 5-year OS were conducted based on risk scores with the “survival” R package (“cph” function) and “rms” R package, by which were visually assessed using calibration plots comparing the predicted and actual survival probabilities among ovarian cancer patients. C-index is the concordance index, which was calculated to estimate the discrimination ability of the signature. The AUC was similar to c-index and a higher value of which indicated better prognostic value.

### Exploration of the relationship between SLC25A10 and prognosis

To calculate the correlation coefficients of SLC25A10 and other genes in TCGA ovarian cancer samples, we employed the spearman method with the R “psych” package, and screened the genes for gene ontology (GO) analysis by the R “clusterProfiler” package [33]. The CellMiner database (https://discover.nci.nih.gov/cellminer/) was used to download drug data and gene expression data [19]. The Pearson correlation coefficients between SLC25A10 expression and different drugs were calculated to reveal the relationship between SLC25A10 expression and drug sensitivity, and the final results were presented by the “ggplot2” and “ggpubr” R packages.

### Statistical analysis.

All statistical analyses in this study were performed by the R software (version 4.0.3). If not otherwise stated, results were considered to be of statistical significance with p < 0.05.

## Data Availability

The datasets used and/or analyzed during the current study are available on the platform of Zenodo (DOI: 10.5281/zenodo.7026755).

## Abbreviations

TCGA: The Cancer Genome Atlas
ICGC: International Cancer Genome Consortium
GTEx: Genotype-Tissue Expression Portal
OS: Overall survival
DEGs: Differentially expressed genes
ROC: Receiver operating characteristic
AUC: Area under the ROC curve
DCA: Decision curve analysis
GO: Gene ontology
